# Non-myeloproliferative Pancytopenia: A Rare Presentation of Thyrotoxicosis

**DOI:** 10.7759/cureus.35076

**Published:** 2023-02-16

**Authors:** Izzathunnisa Rahmathullah, Maheswaran Umakanth, Suranga Singhapathirane

**Affiliations:** 1 General Medicine, Teaching Hospital Batticaloa, Batticaloa, LKA; 2 Clinical Medicine, Eastern University, Sri Lanka, Colombo, LKA; 3 Medicine, Teaching Hospital Batticaloa, Batticaloa, LKA

**Keywords:** carbimazole, tpo antibody, rare presentation, pancytopenia, thyrotoxicosis

## Abstract

Thyrotoxicosis-induced pancytopenia is a rare manifestation. The practical challenge is to differentiate thyrotoxicosis-induced pancytopenia from the side effects of anti-thyroid treatment following the commencement of treatment. Although some of the possible underlying pathogeneses have been reported, the complete mechanisms remain unclear concerning pancytopenia in uncontrolled thyrotoxicosis. Thyrotoxicosis-induced pancytopenia is completely reversible with the administration of anti-thyroid drugs at an appropriate time and regular follow-up to prevent further recurrence.

## Introduction

Pancytopenia is one of the common clinical conditions seen in daily medical practice. There are several causes for pancytopenia ranging from simple viral infection to bone marrow suppression. Thyrotoxicosis is also one of the rare differential diagnoses for pancytopenia. Although isolated anemia, neutropenia, or thrombocytopenia is a well-known hematological manifestation of uncontrolled thyrotoxicosis, pancytopenia without myeloproliferative transformation is a rare clinical entity of thyrotoxicosis [1].

Correct diagnosis and appropriate treatment are the mainstays of recovery. Most anti-thyroid drugs also induce pancytopenia which confuses the diagnosis of thyrotoxicosis-induced pancytopenia. Here, we discuss the case of a patient who presented with thyrotoxicosis-induced pancytopenia which resolved completely with appropriate anti-thyroid drugs along with improved thyroid functions back to normal.

## Case presentation

A 66-year-old woman was admitted to our unit in March 2022 with complaints of difficulty in breathing, chest pain, and palpitation for one week. Her medical history included type 2 diabetes for 10 years with microvascular complications. She had a significant weight loss of around 6 kg in the last two months despite having a good appetite. She also had increased sweating and frequency of diarrhea. She denied any bleeding manifestations. She was a lifelong non-smoker and a non-drinker. She had been taking gliclazide, aspirin, and atorvastatin regularly for the past seven years.

On presentation, she looked ill but afebrile. She was pale, mildly icteric, and had exophthalmos. There was no lymphadenopathy. Physical examination revealed sweating in both palms without any evidence of clubbing in the fingernails. Her heart rate was 120 beats per minute with a regular rhythm, and her blood pressure was 150/90 mmHg with a respiratory rate of 20 cycles per minute. Initially, she maintained a saturation of 96% with 2 L of face mask oxygen and later stabilized on room air.

Her thyroid gland was diffusely enlarged, firm, warm, non-tender, and without any bruit. No breast lumps were identified. Her apex was located in the sixth intercostal space in the midclavicular line, and her heart sounds were normal without any murmur. Lung fields were clear with bilateral equal breath sounds. The abdomen was soft, and no hepatosplenomegaly or free fluid was noted. The rest of her system examinations were unremarkable. The detailed relevant blood investigations are presented in Table 1.

**Table 1:** Relevant investigations of the patient on admission.

Clinical parameters	Patient’s value	Reference value
Fasting blood sugar (mg/dL)	143	99–125
Random blood sugar (mg/dL)	180	140–200
White blood cells (/μL)	2,086	4,000–10,000
Hemoglobin (g/dL)	7.0	13–15
Platelets (/μL)	90,000	130,000–400,000
Erythrocyte sedimentation rate (mm/1^st^ hour)	51	0–22
C-reactive protein (mg/dL)	14	0.2–3
Lactate dehydrogenase (U/L)	180	81–234
Direct agglutination test	Negative	
Reticulocyte count (%)	1.1	<2
Thyroid-stimulating hormone (µIU/L)	0.015	0.4–4
FreeT4 (pmol/L)	83.5	9–23
T3 (ng/ mL)	4.65	0.8–2.0
Thyroxine peroxidase antibody (IU/mL)	1,003	<35
Serum ferritin (ng/mL)	184	11.1–264
Serum iron (μmol/L)	8.2	6.6–30.4
Total iron-binding capacity (μmol/L)	49.5	47.4–89
Iron saturation (%)	20	15–45
Corrected calcium (mmol/L)	2.3	2.2–2.6
Serum sodium (mmol/L)	138	135–145
Serum potassium (mmol/L)	4.0	3.5–4.5
Aspartate transferase (IU/L)	62	8–33
Alanine transaminase (IU/L)	55	4–36
Prothrombin time/International normalized ratio	1.24	<1.5
Total bilirubin (µmol/L)	18	1.71–20.5
Blood urea (mmol/L)	8.5	1.3–6.3
Serum creatinine (μmol/L)	79	53–88

The blood picture was reported as pancytopenia without any evidence of malignancy or ongoing hemolysis. Her retroviral studies and viral hepatitis screening were unremarkable. Thyroid-stimulating hormone receptor antibody was not done due to a lack of resources. Blood and urine culture was negative for pathogens. Stool occult blood was negative. Urine full report was normal except for albuminuria, which was suggestive of diabetic nephropathy.

**Figure 1:**
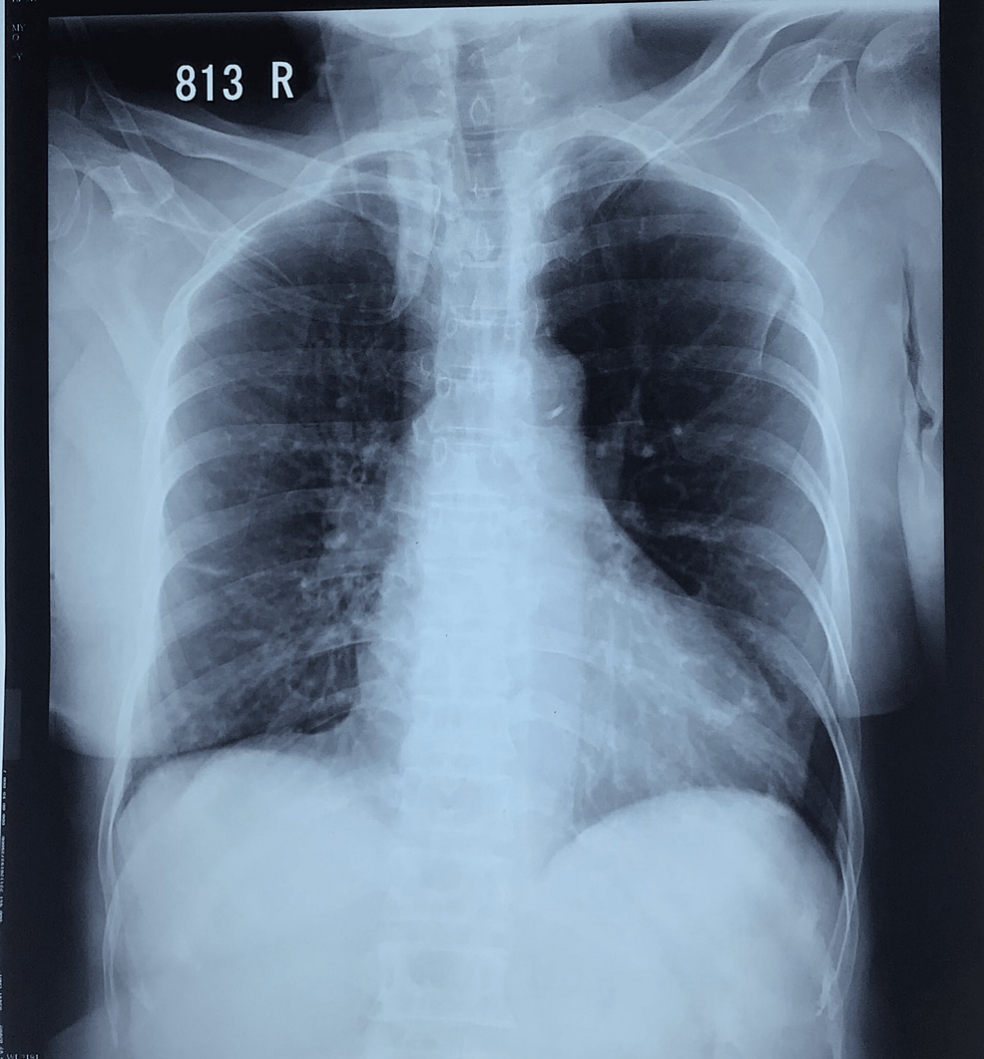
The chest X-ray showing early pulmonary edema.

**Figure 2:**
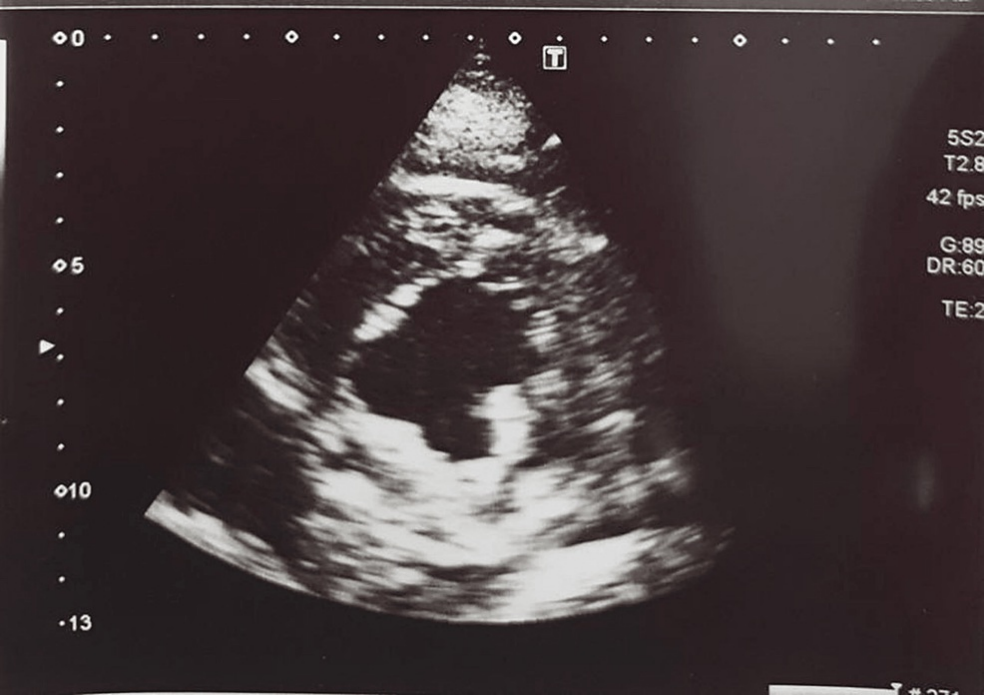
The two-dimensional echocardiogram showing mild left ventricular dysfunction.

The chest X-ray revealed early pulmonary edema and the two-dimensional echocardiogram showed mild left ventricular dysfunction with an ejection fraction of 50% (Figures 1, 2).

The upper gastrointestinal endoscopy was reported as normal and did not reveal any significant growths, ulcers, or bleeding points.

**Figure 3:**
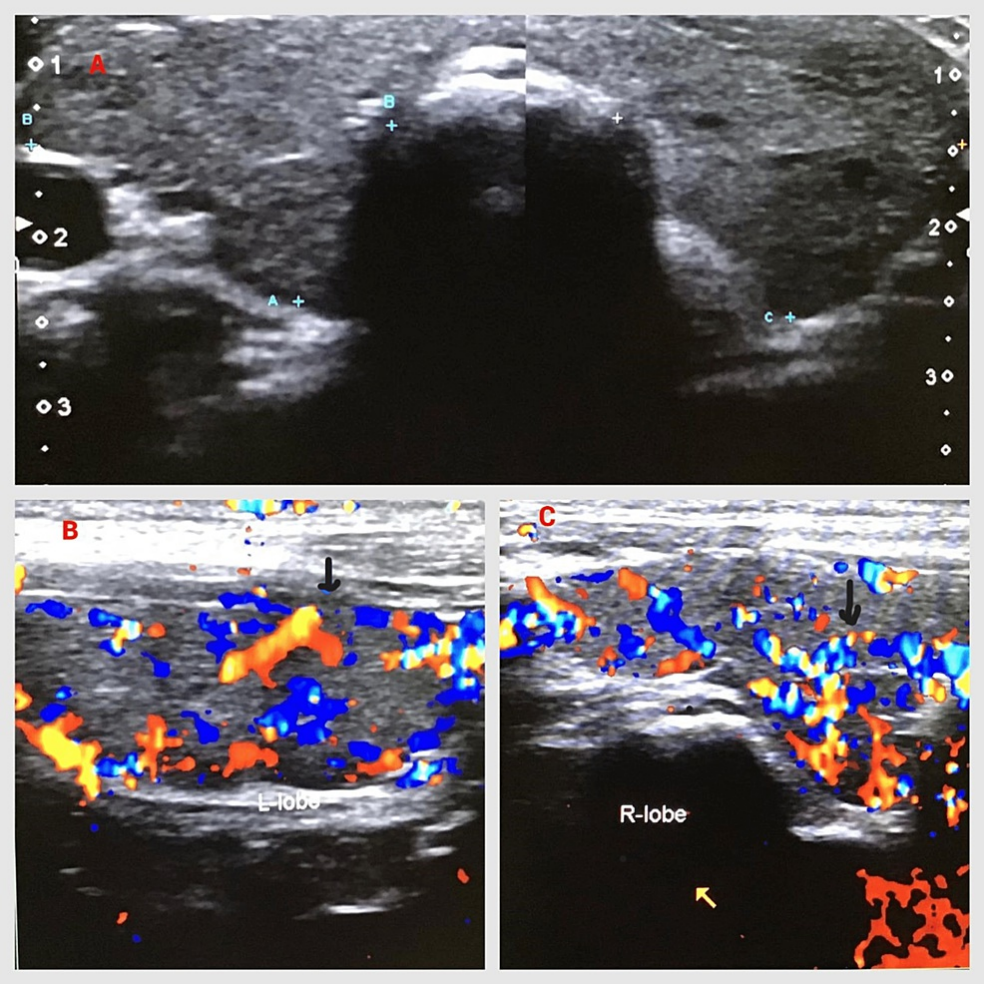
Ultrasound scan neck of the thyroid gland with the Doppler effect. A: Thyroid goiter with diffusely enlarged lobes. B, C: Left and right thyroid lobes with increased vascularity, suggestive of thyroiditis. Black arrows point to the higher vascular areas.

The ultrasound scan of the abdomen and pelvis was unremarkable. The ultrasound scan of the neck revealed a diffusely enlarged goiter with increased vascularity, suggestive of thyroiditis (Figures 3A-3C).

She underwent a bone marrow biopsy which showed hypercellularity with adequate megakaryocyte and hyperplasia of both myeloid and erythroid series.

Under the impression of Graves’ disease disease with severe hyperthyroidism, we started anti-thyroid treatment with carbimazole 10 mg three times per day and lithium carbonate with the liaison of the endocrinology team. We continuously monitored her investigations, particularly the full blood count associated with her signs and symptoms since the treatment started. She was symptomatically managed with propranolol for heart rate control as well as two packs of red cell concentrations were transfused for anemia and a short course of furosemide 40 mg two times per day.

She improved clinically during the hospital stay with the treatment, and we could stop lithium carbonate slowly. Her blood counts and free T4 thyroxin level also started to improve gradually (Tables 2, 3).

**Table 2:** Follow-up hematological values with progressive response to treatment.

Hematological values	Day 1	Day 5	Day 7	Day 9	Day 11
Leukocytes (×1,000/µL)	2.08	2.11	3.1	3.5	4.7
Hemoglobin (g/dL)	7.0	7.4	8.2	10.7	11.2
platelets (×1,000/µL)	90	92	95	117	135

**Table 3:** Follow-up thyroid function tests with gradual response to anti-thyroid treatment.

Thyroid function values	Day 1	Day 14	Day 21
Thyroid-stimulating hormone (µIU/L)	<0.015		<0.015
Free T4 ( pmol/L)	83.5	55.5	31.2
T3 (ng/mL)	4.65		3.0

After three weeks, she was discharged without any significant symptoms and was followed up at the clinic level monthly. Her repeated full blood counts were normal in three cell lines. She was asked to continue carbimazole regularly and adhere to the other routine drugs. Regular thyroid function tests were arranged for further monitoring at the clinic level.

## Discussion

As our patient presented with features of hyperthyroidism with significant weight loss, tremors, chest pain, and shortness of breath and was found to have diffuse goiter with exophthalmos and tachycardia, one of our main differential diagnoses was thyrotoxicosis clinically. Considering the investigations, pancytopenia was noted and extensively excluded other possible causes of pancytopenia such as malignancies, recent-onset viral fever, or any other infections, medication-induced pancytopenia, as well as hemolysis. The remaining investigations including blood pictures and bone marrow biopsy excluded other possibilities. Her thyroid function test showed thyrotoxicosis. Considering thyrotoxicosis-induced pancytopenia, we started treatment with carbimazole regularly after liaison with the endocrinology team [1,2]. She gradually started to respond. At the same time, her three cell lines also showed significant improvement, ultimately confirming our diagnosis.

A rare presentation of thyrotoxicosis is pancytopenia and its related symptoms. Even though it was reported as anemia, leukopenia, or thrombocytopenia individually, a combined presentation of thyrotoxicosis-induced pancytopenia was reported as a rare manifestation [1,2]. It has been mentioned that thyrotoxicosis-induced anemia is the most commonly associated presentation (10-34%), followed by leukopenia (15-30%). Only 2-5% have reported thyrotoxicosis-induced thrombocytopenia [1,2].

Although the underlying mechanisms are unclear, several factors have been identified as the underlying pathologies. Excess thyroid hormone secretion and its toxicity to bone marrow cells cause functional hyperactivity of reticuloendothelial cells, leading to ineffective erythropoiesis. This hyperproliferation of immature erythroid cells and its sequestration dramatically increases all types of anemia by exaggerated intake of iron, folic acid, and vitamin B12 [2].

Only two important cases have been reported with severe vitamin B12 deficiency [2-4]. Additionally, reduced red cell life span also contributes to anemia [5]. The association between hyperthyroidism and splenomegaly has been reported in several places, which also explains the reduced life span of red cells here [6,7]. However, the spleen returns to its normal size once the euthyroid state is achieved [8]. Some autoimmune processes are also suspected of gradually affecting the bone marrow stem cells due to excess hormone secretion [9,10]. According to the literature, 22% of Graves’ disease patients have anemia [11,12].

Around 30% of patients develop leukopenia in hyperthyroidism [1,2]. Mainly two underlying pathologies have been suggested. Reduced marrow granulocyte reserve restricts effective granulopoiesis [3]. A widespread antigenicity reaction between thyroid-stimulating hormone receptors and polymorphonuclear neutrophils destruct the white cells [13]. A relative lymphocytosis with normal or mildly reduced white cells that characterize hyperthyroidism is known as Kocher’s blood picture [6].

Hyperthyroidism-induced thrombocytopenia has been explained via several mechanisms. Antiplatelet antibodies were detected in around 50% of patients with Graves’ disease [2,3]. Functional hypersplenism-induced thrombocytopenia is also a detectable factor in reduced platelet count [4].

All patients in the above cases were successfully treated with anti-thyroid drugs and recovered fully. Thyrotoxicosis-induced pancytopenia needs to be considered as one of the differential diagnoses in patients who present with unexplained pancytopenia and needs to be treated promptly. For appropriate treatment, thyrotoxicosis-induced pancytopenia should be differentiated from drug-induced pancytopenia in hyperthyroid patients.

## Conclusions

Pancytopenia is one of the rare manifestations of thyrotoxicosis. Anti-thyroid drugs are the cornerstone of therapy, and, once diagnosed, a full recovery may be possible. Patients who present with unexplained pancytopenia and require immediate treatment should be evaluated for thyrotoxicosis-induced pancytopenia as one of the differential diagnoses. Thyrotoxicosis-induced pancytopenia in hyperthyroid patients should be distinguished from drug-induced pancytopenia in the interim for proper therapy.

## References

[1] Kim TH, Yoon JS, Park BS (2013). A case of pancytopenia with hyperthyroidism. Yeungnam Univ J Med.

[2] Lima CS, Zantut Wittmann DE, Castro V, Tambascia MA, Lorand-Metze I, Saad ST, Costa FF (2006). Pancytopenia in untreated patients with Graves' disease. Thyroid.

[3] Pincet L, Gorostidi F (2018). Graves disease causing pancytopenia: case report and literature review. Clin Med Insights Case Rep.

[4] Heng LH, Tan F (2013). Pancytopenia in a patient with Grave's disease. Med J Malaysia.

[5] Duquenne M, Lakomsky D, Humbert JC, Hadjadj S, Weryha G, Leclère J (1995). [Pancytopenia resolved by the treatment of hyperthyroidism]. Presse Med.

[6] Garcia J, França Ld, Ellinger V, Wolff M (2014). Marrow hypoplasia: a rare complication of untreated Grave's disease. Arq Bras Endocrinol Metabol.

[7] Soeki T, Tamura Y, Kondo N, Shinohara H, Tanaka H, Bando K, Fukuda N (2001). A case of thyrotoxicosis with pancytopenia. Endocr J.

[8] Chen YH, Lin HJ, Chen KT (2009). Rare presentations of hyperthyroidism--Basedow's paraplegia and pancytopenia. Am J Emerg Med.

[9] Weitzman SA, Stossel TP, Harmon DC, Daniels G, Maloof F, Ridgway EC (1985). Antineutrophil autoantibodies in Graves' disease. Implications of thyrotropin binding to neutrophils. J Clin Invest.

[10] Perlman JA, Sternthal PM (1983). Effect of 131I on the anemia of hyperthyroidism. J Chronic Dis.

[11] Gianoukakis AG, Leigh MJ, Richards P (2009). Characterization of the anaemia associated with Graves' disease. Clin Endocrinol (Oxf).

[12] Shin JH, Kim HJ, Kim SB (2009). A case of Graves’ disease with pancytopenia. J Korean Endocr Soc.

[13] Adrouny A, Sandler RM, Carmel R (1982). Variable presentation of thrombocytopenia in Graves' disease. Arch Intern Med.

